# Multidrug-resistant enteric pathogens in older children and adults with diarrhea in Bangladesh: epidemiology and risk factors

**DOI:** 10.1186/s41182-021-00327-x

**Published:** 2021-05-10

**Authors:** Stephanie C. Garbern, Tzu-Chun Chu, Monique Gainey, Samika S. Kanekar, Sabiha Nasrin, Kexin Qu, Meagan A. Barry, Eric J. Nelson, Daniel T. Leung, Christopher H. Schmid, Nur H. Alam, Adam C. Levine

**Affiliations:** 1 Department of Emergency Medicine, Alpert Medical School, Brown University, 55 Claverick, 2nd Floor, Providence, RI 02903 USA; 2 Center for Statistical Sciences, Brown University, 121 South Main Street, Providence, RI 02903 USA; 3 Rhode Island Hospital, 593 Eddy St, Providence, RI 02903 USA; 4 Brown University, 69 Brown St, Providence, RI 02906 USA; 5 International Centre for Diarrhoeal Disease Research, Bangladesh (icddr,b), 68 Shaheed Tajuddin Ahmed Ave, Dhaka, 1212 Bangladesh; 6 Department of Biostatistics, Brown University School of Public Health, 121 South Main Street, Providence, RI 02903 USA; 7 Departments of Pediatrics and Environmental and Global Health, Emerging Pathogens Institute, University of Florida, 2055 Mowry Road, Gainesville, FL 32610 USA; 8 Division of Infectious Diseases, University of Utah School of Medicine, 30 N 1900 E, Room 4B319, Salt Lake City, UT 84132 USA

**Keywords:** Antimicrobial resistance, Multidrug resistance, Global health, Diarrhea, Low- and middle-income countries, Resource-limited, Enteric pathogens, Bangladesh

## Abstract

**Background:**

Antimicrobial resistance (AMR) is a global public health threat and is increasingly prevalent among enteric pathogens in low- and middle-income countries (LMICs). However, the burden of multidrug-resistant organisms (MDROs) in older children, adults, and elderly patients with acute diarrhea in LMICs is poorly understood. This study’s aim was to characterize the prevalence of MDR enteric pathogens isolated from patients with acute diarrhea in Dhaka, Bangladesh, and assess a wide range of risk factors associated with MDR.

**Methods:**

This study was a secondary analysis of data collected from children over 5 years, adults, and elderly patients with acute diarrhea at the International Centre for Diarrhoeal Disease Research, Bangladesh Dhaka Hospital between March 2019 and March 2020. Clinical, historical, socio-environmental information, and a stool sample for culture and antimicrobial susceptibility testing were collected from each patient. Univariate statistics and multiple logistic regression were used to assess the prevalence of MDR among enteric pathogens and the association between independent variables and presence of MRDOs among culture-positive patients.

**Results:**

A total of 1198 patients had pathogens isolated by stool culture with antimicrobial susceptibility results. Among culture-positive patients, the prevalence of MDR was 54.3%. The prevalence of MDR was highest in *Aeromonas* spp. (81.5%), followed by *Campylobacter* spp. (72.1%), *Vibrio cholerae* (28.1%), *Shigella* spp. (26.2%), and *Salmonella* spp. (5.2%). Factors associated with having MDRO in multiple logistic regression included longer transport time to hospital (>90 min), greater stool frequency, prior antibiotic use prior to hospital presentation, and non-flush toilet use. However, pseudo-R2 was low 0.086, indicating that other unmeasured variables need to be considered to build a more robust predictive model of MDR.

**Conclusions:**

MDR enteric pathogens were common in this study population with clinical, historical, and socio-environmental risk factors associated with MDROs. These findings may help guide clinical decision-making regarding antibiotic use and selection in patients at greatest risk of complications due to MDROs. Further prospective research is urgently needed to determine what additional factors place patients at greatest risk of MDRO, and the best strategies to mitigate the spread of MDR in enteric pathogens.

**Supplementary Information:**

The online version contains supplementary material available at 10.1186/s41182-021-00327-x.

## Background

Diarrheal diseases are a leading cause of morbidity and mortality worldwide, causing over 6.3 billion episodes and 1.3 million deaths annually, with the vast majority of cases occurring in low- and middle-income countries (LMICs) [1, 2]. While the majority of diarrhea cases are self-limiting, and the mainstay of treatment is rehydration, antibiotics are recommended by the World Health Organization (WHO) for treatment of certain pathogenic causes of diarrhea [3]. For example, treatment of patients with *Vibrio cholerae* (*V. cholerae*) with severe dehydration and Shigellosis is recommended to reduce the duration of symptoms and patient-to-patient transmission [3]. However, for other etiologies of diarrhea, antibiotics are generally not indicated [3]. Ideally, decisions regarding antimicrobial use should be guided by microbiological testing such as stool culture with susceptibilities or molecular diagnostics; however, these tests are unavailable in the vast majority of LMIC clinical settings [4, 5]. This lack of diagnostic testing availability together with shortages of healthcare providers to guide antibiotic use has often led to long-standing practices of antibiotic overuse, contributing to high antimicrobial resistance (AMR) rates among enteric pathogens in LMICs. Additionally, clinicians often make decisions regarding antibiotic use and selection based on syndromic guidelines and limited local susceptibility patterns with minimal consideration of individual risk factors for AMR [3, 5].

AMR has been identified by the World Health Organization (WHO) as a serious global public health concern that must be addressed with urgency [6]. In LMICs, AMR rates of various enteric pathogens have been increasing due to a multitude of reasons including widespread availability and unregulated sale of antibiotics, poor drug quality assurance, long-standing patient expectations for antibiotics, and limited public health knowledge of AMR [6–8]. Alarmingly, an AMR surveillance study in Nepal showed an increase in MDR to 100% among *V. cholerae* samples during the 2006-2016 period [9]. Furthermore, emerging multi-drug resistance (MDR) in LMICs threatens to limit the efficacy of commonly used and low-cost antimicrobials. Individuals with MDR infections are more likely to have longer hospital admissions, higher healthcare costs, prolonged time to recovery, and higher case fatality [6]. MDR infections not only negatively impact individuals but also create substantial challenges for clinicians and healthcare systems [6]. Despite the extent of the problem, AMR and especially the burden of multidrug-resistant organisms (MDRO) are very poorly understood in LMICs, with patterns of resistance fluctuating greatly between regions and over time, even within the same country [7, 10].

While several large recent studies, such as the Global Enteric Multicenter Study, have investigated the etiologies of diarrhea in children under 5 years of age in LMICs, there is a stark lack of data for older children, adolescents, adults, and the elderly [11]. Diarrheal etiologies may vary greatly among these age groups in whom a substantial burden of disease exists, with patients over 70 years constituting more than 40% of all global deaths due to diarrhea in 2016 [12]. Understanding the epidemiology and risk factors associated with MDR in common enteric pathogens among older children, adults, and elderly individuals with diarrhea can lead to more evidence-based decision-making regarding testing and treatment of patients at highest risk for complicated disease. The aim of this study was to characterize the epidemiology of MDR enteric pathogens isolated from patients over 5 years old with acute diarrhea in Dhaka, Bangladesh, and determine the clinical, historical, and socio-environmental risk factors associated with MDR. This information may be beneficial in guiding clinicians and public health practitioners in managing patients with acute diarrhea as well as reducing the impact of MDR on communities.

## Methods

### Study design

This was a secondary analysis of data collected from the “Novel, Innovative Research for Understanding Dehydration in Adults and Kids” (NIRUDAK) study, a prospective cohort study of patients over 5 years presenting with acute diarrhea to the rehydration unit at Dhaka Hospital of the International Centre for Diarrhoeal Disease Research, Bangladesh (icddr,b) [13]. Ethical approval for the NIRUDAK Study was obtained from the icddr,b Ethical Review Committee and Rhode Island Hospital Institutional Review Board. Data from the NIRUDAK study was obtained through the NIRUDAK Principal Investigator (ACL; last author on this study). Data for the present study consisted only of de-identified data with all personal identifiers removed, and therefore ethical approval for this secondary analysis was not necessary.

### Study setting and population

The NIRUDAK study was conducted between March 2019 and March 2020 at icddr,b Dhaka Hospital, an urban referral hospital that provides free clinical services for over 100,000 patients annually. The inclusion and exclusion criteria used for the present study were the same as the parent (NIRUDAK) study. All patients over 5 years of age with acute diarrhea (using the World Health Organization definition of diarrhea as three or more loose stools in the past 24 h lasting less than 7 days) were eligible for enrollment [3, 14]. Exclusion criteria included the following: having less than three loose stools in the past 24 h, diarrhea lasting more than 7 days, a clear alternative diagnosis to gastroenteritis, and previous enrollment in the study. Research staff provided patients and/or their guardian with information about the risks, and benefits of the study and obtained verbal/written consent in the local language, Bangla. In cases where the patient or legal guardian could not read or write, research staff obtained verbal consent asked the parent or guardian to mark the consent form with a thumbprint. For children over the age of 8 years and under the age of 18, verbal or written assent was also obtained.

### Study procedures

Enrolled subjects were clinically assessed by a study nurse for clinical signs and symptoms, historical, demographic, and socio-environmental data. Socio-environmental data included the following: monthly household income, highest education level obtained by participant or parent, water source, use of treated water, toilet facility type, number of people sharing waste facilities, number of people living in household and transport time to hospital. All baseline clinical data were obtained from either the patient and/or parent/guardian and recorded on a case report form. Study procedures were not allowed to delay emergent care, such as placing an intravenous line or delivering fluids. After initial assessment, patients were treated according to standard icddr,b protocols for management of acute diarrhea and per physician discretion including oral or intravenous rehydration and antibiotics. Percent weight change with rehydration was used as the criterion standard for percent dehydration [15, 16]. Patients were categorized as having severe (>9%) dehydration, some (3–9%) dehydration, or no (<3%) dehydration [14]. Prior antibiotic use was determined by patient/parent report of any prior medications used (including antibiotics) before hospital evaluation; those who answered “don’t know” in response to the question on prior medication use were coded as “non-antibiotic” use for the purpose of this analysis.

### Laboratory data and microbiological evaluation

Two stool specimens (at least 2 ml/vial) were collected from each subject—one for analysis to the clinical microbiology laboratory and one for storage in 70% ethanol. Each specimen was screened for common enteric pathogens using stool culture. Isolation, identification, serogrouping, and biotyping of stool samples were performed using standard procedures [17]. Briefly, *V*. *cholerae* was isolated by growth on tellurite taurocholate gelatin agar (TTGA) media with enrichment in bile peptone broth. *Salmonella* spp. and *Shigella* spp. were isolated by growth on MacConkey agar and Salmonella-Shigella agar with enrichment in selenite broth followed by antisera panel testing (Denka Seiken, Tokyo, Japan), *Campylobacter* spp. were isolated by growth on Brucella *agar*, and *Aeromonas* spp. were isolated by growth on TTGA and gelatin agar followed by phenotypic characterization of long-sugar metabolism. Antimicrobial susceptibility testing (AST) was determined by the Kirby-Bauer standard disk diffusion method on Muller–Hinton agar. The results were reported as sensitive, intermediate, and resistant by a method based on the cutoff of the zone size for different antibiotics according to the latest available Clinical and Laboratory Standards Institute guidelines [18].

Pathogens resistant to at least one agent in ≥3 antimicrobial categories were defined as MDR based on consensus definitions from the European Centre for Disease Prevention and Control and the Centers for Disease Control and Prevention [19]. Isolates with a result of “intermediate” were grouped with those with a result of “resistant” for the purpose of this analysis. Aminoglycosides included amikacin and gentamycin; first/second-generation cephalosporins included cefoxitin, cefuroxime; third-generation cephalosporins included cefotaxime, cefixime, ceftazidime, and ceftriaxone; quinolones included ciprofloxacin and nalidixic acid; pencillins included amoxicillin, ampicillin, ticarcillin-clavulanic acid, mecillinam, piperacillin-tazobactam; tetracyclines included doxycycline and tetracycline; sulfonamides included trimethoprim-sulfamethoxazole; colistin and chloramphenicol were also included in AST.

### Statistical analysis

Categorical variables were described using frequencies with percentages. Continuous variables with normal distribution were presented as means with standard deviations (SD). Variables were also stratified by age group (child, adult, elderly) in supplementary analysis (Additional file 1). Comparisons across age groups were conducted with one-way analysis of variance, Pearson’s chi-squared test, or Fisher’s exact test as appropriate. Bivariate analyses evaluated differences between those with and without presence of MDR enteric pathogens, with magnitudes of effect given as odds ratios (OR) and their respective 95% confidence intervals (CI). Multiple logistic regression analysis was performed to identify clinical, historical, and socio-environmental variables independently associated with MDR enteric pathogens with results expressed as adjusted odds ratios (aORs) and their respective 95% CIs. All candidate variables based on expert judgment were retained in the multiple regression analysis as this was an exploratory study with the aim of evaluating potential associations with MDR rather than the creation of a new prediction model. Additionally, due to the large number of observations, the study met a general rule of thumb from the literature of having at least 10 events per candidate variable [20]. Continuous variables were recoded as binary variables by using the median value of the distribution or clinically relevant cutoff points. Nagelkerke’s pseudo-*R*^2^, a measure analogous to the *R*^2^ used in logistic regression, was calculated to provide a global measure of the estimated explained variance of the final model on a new data set; the pseudo-*R*^2^ ranges between 0 and 1 where 1 is a fully explained model [21].

Two sensitivity analyses were conducted. As there remains significant uncertainty regarding the etiologic role of *Aeromonas* spp. as an enteric pathogen in diarrheal disease, an analysis was performed in which samples with isolation of *Aeromonas* spp. only were excluded [22, 23]. In the second sensitivity analysis, patients who had reported “don’t know” regarding prior medications taken were excluded from analysis. For all analyses, a two-tailed *p* value of 0.05 was considered statistically significant. STATA Version 14 (Stata Corp; College Station, USA) and *R* (R Foundation for Statistical Computing, Vienna, Austria) were used for all analyses.

## Results

### Enrollment and baseline characteristics

**Fig. 1:**
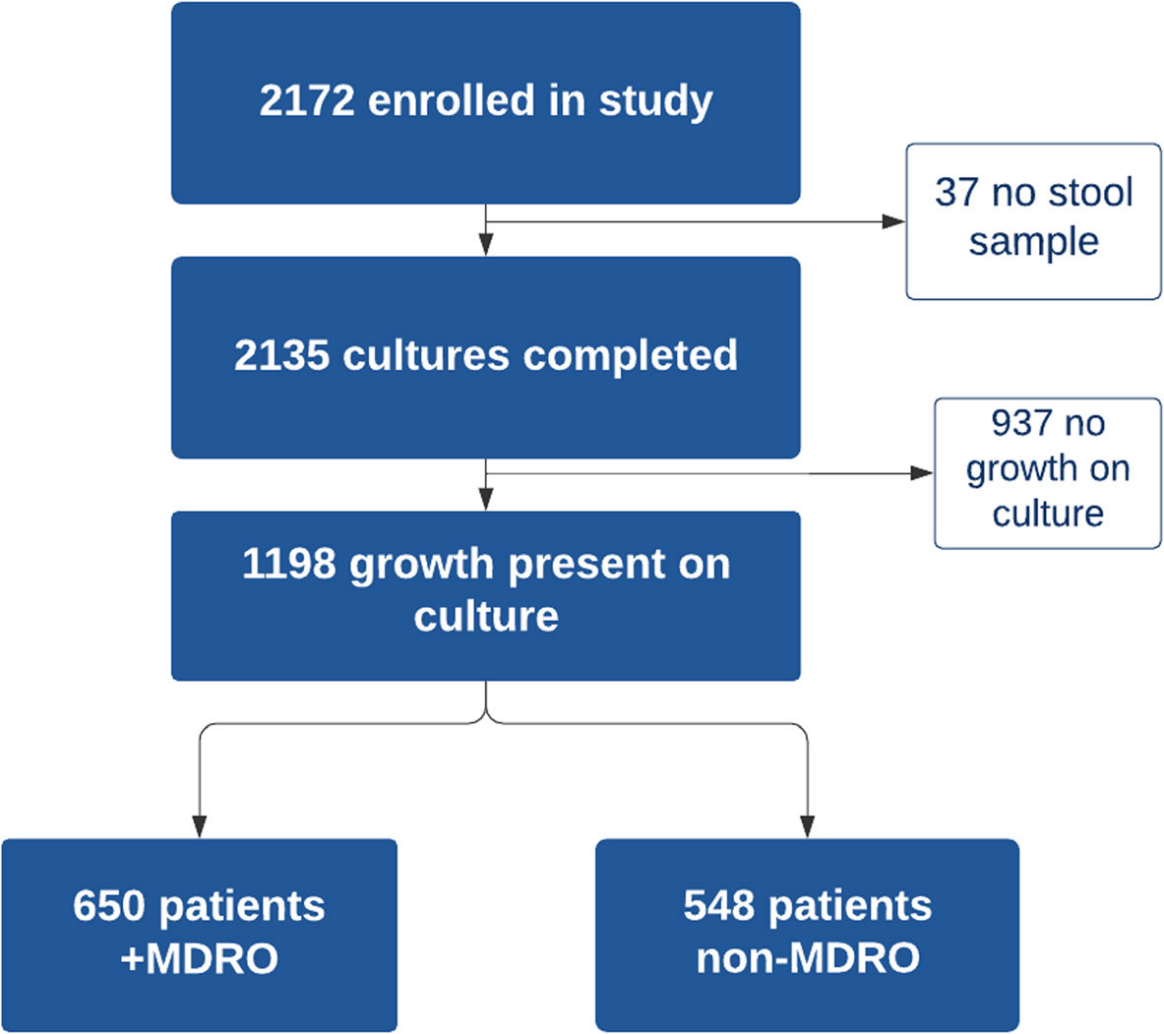
Flowchart of included study patients

During the study period, 2172 patients ≥ 5 years of age with acute diarrhea presenting to icddr,b Dhaka Hospital were enrolled in the NIRUDAK study. Stool culture was completed for 2135 patients, with 1198 samples (56.1%) having pathogens isolated on stool culture (“positive culture”). For the present analysis, only records with growth on stool culture were included for further analysis as shown in Fig. 1. The median age of the study population was 30 years (IQR, 17-60 years; range, 5-100 years), and 577 (48.2%) patients were female.

Of the samples with growth on stool culture, the majority (1025 or 85.6%) had a single bacterial pathogen isolated. The prevalence of enteric pathogens isolated from stool samples of the study population are shown in Table 1. “Other” organisms were isolated in 13 samples (1.1%) and included *Vibrio fluvialis*, *Vibrio parahaemolyticus*, and *Plesiomonas shigelloides.* In 173 samples (14.4%), there was more than one bacterial pathogen isolated.

**Table 1:** Prevalence of enteric pathogens isolated from stool samples

	(***n***=1198) ***n*** (%)
**Pathogens isolated**	
*Vibrio cholerae* total	509 (42.5)
O1	493 (41.2)
O139	0 (0)
Non-O1/O139	16 (1.3)
*Shigella* spp. total	24 (2.0)
*S. boydii*	0 (0)
*S. dysenteriae*	2 (0.2)
*S. flexneri*	17 (1.4)
*S. sonnei*	5 (0.4)
Other/undefined	2 (0.2)
*Campylobacter* spp. total	76 (6.3)
*Aeromonas* spp. total	363 (30.3)
*A. caviae*	5 (0.4)
*A. hydrophila*	247 (20.6)
*A. sobria*	41 (3.4)
Other/undefined	70 (5.8)
*Salmonella* spp*.* total	40 (3.3)
Serogroup A	0 (0)
Serogroup B	19 (1.6)
Serogroup C	12 (1.0)
Serogroup D	2 (0.2)
Serogroup E	3 (0.3)
Other/undefined	2 (0.2)
Other organisms total	13 (1.1)
**Multiple pathogens total**^**a**^	173 (14.4)

^a^“Multiple pathogens” indicates detection of ≥ 2 organisms on either stool culture (*Vibrio cholerae*, *Shigella* spp., *Campylobacter* spp., *Aeromonas* spp., *Salmonella* spp., or other bacterial organism). All other lines in the table indicate isolation of the single organism listed (i.e., no co-infections present)

The antibiotic resistance pattern of isolated pathogens by antibiotic category and the prevalence of MDR by pathogen type are shown in Table 2. A total of 650 (30.4%) study patients had MDRO isolated from stool samples, representing 54.3% of patients with positive cultures. The prevalence of MDR was highest in *Aeromonas* spp. (81.5%), followed by *Campylobacter* spp. (72.1%), *V. cholerae* (28.2%), *Shigella* spp. (26.2%), and *Salmonella* spp. (5.2%).

**Table 2:** Antimicrobial resistance patterns of stool isolates

	AMG	CEF	CRB	CHL	FQ	GLY	MAC	PCN	TET	TMS	MDR
	*n* (%)										
*Vibrio cholerae* (*n=*641)	0	12 (1.9)	-	-	7 (1.1)	1 (0.2)	633 (98.8)	181 (28.2)	9 (1.4)	626 (97.7)	185 (28.9)
*O1* (*n=*623)	0	9 (1.4)	-	-	1 (0.2)	0	618 (99.2)	178 (28.6)	2 (0.32)	615 (98.7)	175 (28.1)
*Non-O1/O139* (*n=*18)	0	3 (16.7)	-	-	6 (33.3)	1 (5.6)	15 (83.3)	3 (16.7)	7 (38.9)	11 (61.1)	10 (55.6)
*Shigella* spp*.* (*n=*42)	0	3 (7.1)	-	-	26 (61.9)	-	11 (26.9)	17 (40.5)	0	22 (52.4)	11 (26.2)
*S. dysenteriae* (*n=*2)	0	0	-	-	0	-	0	1 (50.0)	0	1 (50.0)	0
*S. flexneri* (*n=*25)	0	0	-	-	16 (64.0)	-	3 (12.0)	8 (32.0)	0	11 (44.0)	4 (16.0)
*S. boydii* (*n=*3)	0	0	-	-	1 (33.3)	-	0	1 (33.3)	0	1 (33.3)	0
*S. sonnei* (*n=*10)	0	3 (30.0)	-	-	9 (90.0)	-	8 (80.0)	6 (60.0)	0	8 (80.0)	7 (70.0)
*Species* (*n=*2)	0	0	-	-	0	-	0	1 (50.0)	0	1 (50.0)	0
*Aeromonas spp.* (*n=*400)	0	2 (0.5)	36 (9.0)	-	292 (73.0)	-	399 (99.8)	69 (17.3)	286 (71.5)	338 (84.5)	326 (81.5)
*A. hydrophila* (*n=*273)	0	2 (0.7)	20 (7.3)	-	220 (80.6)	-	272 (99.6)	42 (15.4)	216 (79.1)	243 (89.0)	235 (86.1)
*A. sobria* (*n=*46)	0	0	6 (13.0)	-	23 (50.0)	-	46 (100.0)	10 (21.7)	27 (58.7)	35 (76.1)	31 (67.4)
*A. caviae* (*n=*5)	0	0	0	-	5 (100.0)	-	5 (100.0)	0	4 (80.0)	5 (100.0)	5 (100.0)
*Undefined* (*species*) (*n=*72)	0	0	10 (13.2)	-	44 (57.9)	-	76 (100.0)	17 (22.4)	39 (51.3)	55 (72.4)	55 (72.4)
*Campylobacter* spp. (*n=*222)	1 (0.5)	143 (64.4)	0	5 (2.3)	205 (92.3)	-	66 (29.7)	134 (60.4)	34 (15.3)	139 (62.6)	160 (72.1)
*Salmonella* spp. (*n=*59)	3 (5.1)	0	0	-	15 (25.4)	-	10 (17.0)	7 (11.9)	-	9 (15.3)	3 (5.1)
*Serogroup A* (*n=*1)	0	0	0	-	1 (100.0)	-	0	1 (100.0)	-	0	0
*Serogroup B* (*n=*24)	0	0	0	-	5 (20.8)	-	6 (25.0)	6 (25.0)	-	4 (16.7)	0
*Serogroup C* (*n=*18)	3 (16.7)	0	0	-	6 (33.3)	-	3 (16.7)	3 (16.7)	-	3 (16.7)	3 (16.7)
*Serogroup D* (*n=*4)	0	0	0	-	3 (75.0)	-	0	0	-	0	0
*Serogroup E* (*n=*5)	0	0	0	-	0	-	0	0	-	1 (20.0)	0
*Undefined species* (*n=*7)	0	0	0	-	0	-	1 (14.3)	1 (14.3)	-	1 (14.3)	0

*AMG* aminoglycoside, *CEF* cephalosporin, *CHL* chloramphenicol, *CRB* carbapenem, *FQ* fluoroquinolone/quinolone, *GLY* glycylcycline, *MAC* macrolide, *PCN* penicillin, *TET* tetracycline, *TMS* trimethoprim-sulfamethoxazole (folate pathway inhibitor), *MDR* multidrug resistant

-, not tested

In bivariate analysis, variables associated with the presence of MDRO included the following: having any sick contacts at home (OR 1.36; 95% CI 1.03-1.79), transport time to hospital > 90 min (OR 1.41; 95% CI 1.06-1.87), greater diarrhea frequency (>10 episodes OR 1.50; 95% CI 1.17-1.93; >20 episodes OR 2.13; 95% CI 1.47-3.09), antibiotic use prior to hospital presentation (OR 1.75, 95% CI 1.38-2.23), female sex (OR 1.26; 95% CI 1.00-1.58), and non-flush toilet use (OR 1.49; 95% CI 1.18-1.87). There was a small but statistically significant difference in temperature between the two groups with patients with MDRO having mean (SD) temperature 0.21 (0.07) degrees Fahrenheit lower than those without MDRO (OR 0.86, 95% CI 0.79-0.95). Results of the bivariate analyses are shown in Tables 3 and 4. Age was not found to be significantly associated with MDRO (OR 1.00, 95% CI 0.99-1.00); when age was categorized as a binary variable (child versus adult), age remained unassociated with MDROs (Table 3). Clinical, historical, and socio-environmental variables and presence of MDROs stratified by age group (child, adult, elderly) are shown in Additional file 1.

**Table 3:** Means of continuous variables and strength of association with multidrug-resistant organisms (MDRO) in bivariate analysis

Characteristic	Overall (***n***=1198)	MDRO (***n***=650) (mean ± SD)	Non-MDRO (***n***=548) (mean ± SD)	OR (95% CI)	***p***
Age (years)	35.9 ± 21.9	35.7 ± 22.2	36.2 ± 21.5	1.00 (0.99-1.00)	0.67
Temperature (F)	97.7 ± 1.2	97.6 ± 1.1	97.9 ± 1.3	0.86 (0.79-0.95)	<0.01
Respiratory rate (breaths/min)	28.7 ± 5.7	28.5 ± 5.5	28.9 ± 6.0	0.99 (0.97-1.01)	0.25
Heart rate (beats/min)	105.2 ± 22.3	104.2 ± 22.1	106.5 ± 22.4	1.00 (0.99-1.00)	0.08
Mean arterial pressure (mmHg)	72.7 ± 15.6	71.9 ± 15.8	73.6 ± 15.4	0.99 (0.99-1.00)	0.05
Mid-upper arm circumference (mm)	234.4 ± 37.6	233.4 ± 37.1	235.6 ± 38.2	0.98 (0.96-1.01)	0.32
% Dehydration	5.9 ± 3.1	5.8 ± 3.0	5.9 ± 3.1	1.00 (0.96-1.04)	0.90
Monthly household income ($100 USD)	1.9 ± 1.3	1.9 ± 1.3	1.9 ± 1.2	0.99 (0.91-1.09)	0.88

Abbreviations: *OR* odds ratio, *CI* confidence interval, *F* Fahrenheit

**Table 4:** Prevalence of categorical variables and association with multidrug-resistant organisms (MDRO) in bivariate analysis

Characteristic	Overall (***N***=1198) ***n*** (%)	MDRO (***N***=650) ***n*** (%)	Non-MDRO (***N***=548) ***n*** (%)	OR (95% CI)	***p***
Age group					
Child (<18 years)	324 (27.1)	182 (28.0)	142 (25.9)	-	0.418
Adult (≥18 years)	874 (73.0)	468 (72.0)	406 (74.1)	0.90 (0.70-1.16)	
Female sex	577 (48.2)	330 (50.8)	247 (45.1)	1.26 (1.00-1.58)	0.049
Altered mental status	63 (5.3)	27 (4.2)	36 (6.6)	0.62 (0.37-1.03)	0.06
Bloody stool reported	13 (1.1)	10 (1.5)	3 (0.6)	2.84 (0.78-10.37)	0.11
Mucoid stool reported	243 (20.3)	133 (20.5)	110 (20.1)	1.02 (0.77-1.36)	0.87
Abdominal pain	515 (43.0)	284 (43.7)	231 (42.2)	1.06 (0.85-1.34)	0.60
Vomiting (>3episodes/24 h)	839 (70.0)	459 (70.6)	380 (69.3)	1.06 (0.83-1.36)	0.63
Diarrhea frequency					<0.01
*≤ 10 episodes/24 h*	405 (33.8)	188 (28.9)	217 (39.6)	-	
*>10 episodes/24 h*	625 (52.2)	353 (54.3)	272 (89.2)	1.50 (1.17-1.93)	
*>20 episodes/24 h*	168 (14.0)	109 (16.8)	59 (10.8)	2.13 (1.47-3.09)	
Prior antibiotic use	435 (36.3)	274 (42.2)	161 (29.4)	1.75 (1.38-2.23)	<0.01
Highest education level					0.34
*No school*	346 (28.9)	181 (27.9)	163 (30.1)	-	
*Primary school*	407 (34.0)	226 (34.8)	181 (33.0)	1.14 (0.85-1.52)	
*Junior secondary*	210 (17.5)	123 (18.9)	87 (15.9)	1.29 (0.91-1.82)	
*Secondary +*	235 (19.6)	120 (18.5)	115 (21.0)	0.95 (0.68-1.33)	
Water source—indoor piped	850 (71.0)	463 (71.2)	387 (70.6)	1.03 (0.80-1.32)	0.82
Use of treated water	498 (41.6)	269 (41.4)	229 (41.8)	0.98 (0.78-1.24)	0.89
Non-flush toilet use	504 (42.1)	302 (46.5)	202 (36.9)	1.49 (1.18-1.87)	<0.01
Sick contacts at home	268 (22.4)	161 (24.8)	107 (19.5)	1.36 (1.03-1.79)	0.03
>9 ppl sharing waste facilities	585 (48.8)	314 (48.3)	271 (49.5)	0.96 (0.76-1.20)	0.69
>5 ppl in household	397 (33.1)	224 (34.5)	173 (31.6)	1.14 (0.89-1.45)	0.29
Time to hospital (>90 min)	258 (21.5)	157 (24.2)	101 (18.4)	1.41 (1.06-1.87)	0.02

Abbreviations: *OR* odds ratio, *CI* confidence interval

-, reference level

All variables except sick contacts at home, gender and temperature remained significant in the multiple logistic regression analysis (Table 5). Generally, the odds ratios were smaller in the multiple regression model with greater diarrhea frequency (>10 episodes OR 1.32, 95% CI 1.01-1.72; >20 episodes OR 1.88; 95% CI 1.26-2.80), transport time to hospital > 90 min (OR 1.43; 95% CI 1.06-1.95), prior antibiotic use (OR 1.70; 95% CI 1.32-2.20), and non-flush toilet use (OR 1.38; 95% CI 1.08-1.78) associated with MDRO. Nagelkerke’s pseudo-*R*^2^ was only 0.086 for the multiple logistic regression model, indicating that the model explained only a small part of the outcome variation.

**Table 5:** Multiple logistic regression analysis of variables and association with multidrug-resistant organisms

Characteristic	aOR	95% CI	***p***
Age (years)	1.00	0.99-1.00	0.26
Female sex	1.19	0.92-1.53	0.18
Temperature (F)	0.90	0.80-1.01	0.08
Respiratory rate (breaths/min)	0.99	0.97-1.01	0.48
Heart Rate (beats/min)	1.00	0.99-1.00	0.17
Mean arterial pressure (mmHg)	1.00	0.99-1.00	0.63
Mid-upper arm circumference (cm)	1.00	0.99-1.00	0.27
% Dehydration	0.98	0.94-1.03	0.45
Altered mental status	0.59	0.34-1.02	0.06
Bloody stool reported	3.41	0.88-13.20	0.08
Mucoid stool reported	0.97	0.72-1.32	0.84
Abdominal pain	1.03	0.81-1.31	0.95
Vomiting (>3episodes/24 h)	0.99	0.75-1.72	0.94
Diarrhea frequency			<0.01
*≤ 10 episodes/24 h*	-	-	
*>10 episodes/24 h*	1.32	1.01-1.72	
*>20 episodes/24 h*	1.88	1.26-2.80	
Prior antibiotic use	1.70	1.32-2.20	<0.01
Monthly household income ($100 USD)	1.00	0.90-1.10	0.96
Highest education level			0.55
*No school*	-	-	
*Primary school*	1.07	0.77-1.51	
*Junior secondary*	1.14	0.76-1.73	
*Secondary +*	0.87	0.57-1.32	
Water source—indoor piped	1.04	0.78-1.39	0.77
Use of treated water	1.01	0.78-1.32	0.93
Non-flush toilet use	1.38	1.08-1.78	0.01
Sick contacts at home	1.27	0.95-1.70	0.10
>5 ppl in household	1.17	0.90-1.51	0.24
Time to hospital (>90 min)	1.43	1.06-1.95	0.02

Abbreviations: *aOR* adjusted odds ratio, *CI* confidence interval, *USD* United States Dollar, *F* Fahrenheit

-, reference level

In sensitivity analysis excluding 363 patients with *Aeromonas* spp. only isolated from culture, multiple logistic regression analysis results were similar with prior antibiotic use (OR 1.91; 95% CI 1.36-2.55) and greater transport time to hospital (OR 1.46; 95% CI 1.01-2.12) remaining significantly associated with MDRO (Additional file 1). However, diarrhea frequency (>10 episodes OR 1.13; 95% CI 0.81-1.58; >20 episodes OR 1.43; 95% CI 0.89-2.32) was no longer found to be associated with MDRO. Additionally, non-flush toilet use was marginally statistically insignificant (OR 1.33; 95% CI 0.98-1.80), while temperature (OR 0.74, 95% CI 0.63-0.87) and mid-upper arm circumference (MUAC) (OR 0.99, 95% CI 0.99-1.00) were negatively associated with MDRO. In the sensitivity analysis (Additional file 2) excluding 199 patients who had reported “don’t know” regarding the type of prior medication used, multiple logistic regression analysis results found the same significant variables as in the main analysis: greater diarrhea frequency (>10 episodes OR 1.47, 95% CI 1.10-1.97; >20 episodes OR 2.08; 95% CI 1.34-3.23), greater transport time to hospital (OR 1.41; 95% CI 1.10-1.98) prior antibiotic use (OR 1.74; 95% CI 1.32-2.29), and non-flush toilet use (OR 1.52, 95% CI 1.15-2.00).

## Discussion

MDR enteric pathogens are an urgent public health threat in LMICs where the largest burden of diarrheal disease persists [24–26]. Such trends are not limited to LMICs, with high-income countries (HICs) also having substantial burdens of MDR, although antimicrobial stewardship efforts, infection control measures, and regulations on antibiotic use among humans and animals have generally been more widely instituted as HICs have more financial and human resources to implement these measures [27, 28]. In this study, over half of all culture-positive samples from this population of patients over 5 years with diarrhea in urban Bangladesh demonstrated MDR. This finding is consistent with a number of other recent studies showing that AMR in enteric pathogens has become commonplace in LMICs [9, 29–31].

Prior antibiotic use was found to be strongly associated with presence of MDROs in this study, similar to findings from prior studies among patients with acute infections from both HICs and LMICs [10]. These results emphasize the important role of assessing an individual’s antibiotic exposure history in determining risk for MDR infections. Antibiotic use was common with 36.3% of patients reporting use of antibiotics for their current illness. Actual antibiotic use is suspected to be even higher as 16% of culture-positive patients reported “I don’t know” and were coded as non-antibiotic in this analysis. These results are consistent with prior studies including a 2019 scoping review of non-prescription antibiotic use in LMICs showing rates from 8% to greater than 90% depending on patients’ level of education, monthly income, and gender [32].

Non-flush toilet use (i.e., pit latrine, open defecation) was associated with MDROs suggesting the important role that improved sanitation systems may play in disrupting cycles of oral-fecal transmission of resistant enteric pathogens, a finding which has been described previously in patients with ciprofloxacin-resistant *Shigella* in Bangladesh [33]. However, handwashing was not found to be associated with MDROs which may be due to limitations in the binary nature of how this question was asked rather than a lack association between personal hygiene practices and risk of MDROs. Prior studies that have evaluated individuals’ self-reported frequency of handwashing (categorized as “never,” “sometimes,” “usually,” or “always”) have shown that lower handwashing frequencies are associated with MDROs [31].

Longer transport time to hospital and greater stool frequency were also significantly associated with MDROs. Patients with longer transport times to hospital, such as those living in semi-urban or rural areas, may have more limited access to healthcare facilities and qualified healthcare providers to guide appropriate antibiotic use. Consequently, they may be more likely to purchase antibiotics directly from local pharmacies or unlicensed/unqualified vendors, potentially leading to higher levels of inappropriate antibiotic use [34]. This explanation is supported by a 2020 study in Bangladesh which found that rural healthcare providers (including qualified practitioners, semi-qualified, and unqualified vendors) had lower awareness of antibiotic resistance and correct antibiotic course durations compared to those in urban areas [35]. Differences in MDR patterns of *Shigella* and *V. cholerae* O1 between rural and urban locations in Bangladesh have also been previously documented and may explain the association between transport time and MDROs [10].

Greater diarrhea frequency, a marker of more severe illness, was found to be significantly associated with MDRO in this study. This may be due to difficult-to-treat MDROs causing more severe illness and symptoms and is consistent with a prior study in Bangladesh showing MDR shigellosis and *V. cholerae* 01 infections exhibited features of more severe illness (including greater stool frequency) compared to antibiotic-susceptible infections [10]. Furthermore, more severe illness may prompt individuals to travel longer distances to the hospital versus seeking care at other closer primary care facilities. The association between transport time to hospital (or distance to hospital, a close correlate) and illness severity has been previously reported in numerous LMIC and HIC settings. For example, a 2011 study of children requiring hospitalization at Kilifi District Hospital in Kenya found that distance to hospital was correlated with disease severity; the authors suspected this was due to families’ being more willing to travel to the hospital for more severe conditions or from delays in care-seeking [36]. Similarly, studies from Burkina Faso and Ethiopia have also shown that child mortality increases with greater transport times to hospital [37, 38]. In the USA, severity of illness has consistently been identified as a key factor for longer travel times to hospital, notably among elderly individuals living in rural areas [39].

Review of the literature shows a lack of consistency in the risk factors assessed in other studies evaluating MDR in enteric pathogens, limiting the ability to compare study findings between different populations. However, prior studies have illustrated that age, gender, and environmental conditions, such as crowding, poor sanitation, and contaminated foods, are highly associated with resistant enteric pathogens [31, 33]. In a study of MDR enteric pathogens among children under 15 years in Kenya, younger children (particularly those < 24 months), HIV exposure, acute malnutrition, and poor sanitation contributed to an increased risk of MDR enteric pathogens [31]. As HIV is much less common in Bangladesh compared to sub-Saharan Africa, HIV status was not measured or assessed in the present study, although HIV may be a significant risk factor for MDR in other populations [31]. Age was not found to be associated with MDR in this population of patients over 5 years old. Reasons for these findings may be due to a consistently high overall use of antibiotics among older children and adults, whereas greater discrepancies in antibiotic use may exist in younger children. A 2020 study from eight LMICs showed that an average of 24 antibiotics prescriptions were given to children by the time they were 5 years old [40]. While malnutrition has been associated with MDR, no association was seen in this study’s primary analysis [31]. This may be due to difficulties in defining malnutrition status among older populations compared to young children, or the role that other chronic comorbidities that affect immune function may play in older individuals relative to the role of malnutrition. However, lower MUAC was associated with MDR in sensitivity analysis excluding *Aeromonas* spp.; this finding may reflect an association between malnutrition states with MDR in other bacterial etiologies such as *V. cholerae* and *Shigella* versus *Aeromonas* spp.

Additionally, while this study found no associations between MDROs and income or education level, this may be due to relative socioeconomic homogeneity in the patient population. Dhaka Hospital, run by a non-profit organization icddr,b, does not charge fees for care, and thus serves a fairly uniformly impoverished population. Lastly, while statistically insignificant in adjusted analysis, an association between female gender and MDR was seen in univariate analysis. Prior studies have cited the influence that gender may have on AMR though differential health-seeking behaviors, educational levels, and antibiotic use between males and females [41].

### Limitations and future directions

This study consisted of data from a single study site in urban Bangladesh and may not be generalizable to other populations given the high variability in resistance patterns between regions [10]. While the objective of this study was not to develop a new predictive model, but rather identify potential risk factors highly associated with MDR to advise further studies, the low pseudo-*R*^2^ statistic suggests a relatively low predictive accuracy. This indicates that other variables, which could not be collected given the nature of this study, should be considered to build a stronger predictive model of MDR. For example, frequency of antibiotic use, prior hospitalizations, and chronic co-morbidities have been shown to be highly associated with MDR in other studies [42].

In countries with large diarrheal burden such as Bangladesh, there may be high rates of asymptomatic bacterial pathogen carriage. This study used stool culture to determine etiologic causes of diarrhea; however, some culture-positive cases may indeed represent asymptomatic colonization. Conversely, culture has low detection rates for certain pathogens, particularly for *Shigella* [43]. Quantitative PCR approaches which consider the baseline levels of asymptomatic carriage may help overcome this limitation [44]. Lastly, while all pathogens were combined in the regression analysis, this may have created heterogeneous categories since different bacterial pathogens may have different mechanisms of non-susceptibility acquisition. Pathogen-specific models should be considered due to the potential difference in associated risk factors for MDR. This dataset was dominated by *V. cholerae* and *Aeromonas* spp.; while sensitivity analysis excluding *Aeromonas* spp. only samples found slight differences in associated risk factors, prior antibiotic use and transport time to hospital remained significantly associated, indicating the broad importance of these factors regardless of pathogen type. Further research defining the role of *Aeromonas* spp. as a significant enteric pathogen in LMICs is greatly needed.

## Conclusion

In order to promote continued cost-effective and judicious use of antimicrobials for diarrhea, it is important to determine characteristics of patients at increased risk of MDROs. This information is beneficial in determining which patients warrant further testing, as well as to guide appropriate antibiotic selection and local antibiotic prescribing guidelines. MDROs were isolated in over half of culture-positive patients in this population over 5 years old in urban Bangladesh, underscoring the extent and need for strategies to reduce AMR in LMICs. Longer transport time to hospital, greater diarrhea frequency, non-flush toilet use, and prior use of antibiotics were all associated with MDROs. These findings highlight the importance of considering patient-specific factors such as access to healthcare facilities, sanitation resources, and practices of antibiotic use in determining individual and population risks of MDR enteric pathogens. Lastly, the pseudo-*R*^2^ of this multiple logistic regression model was low indicating that further prospective research is urgently needed to explore additional factors that may place patients at greatest risk of MDRO.

## Supplementary Information

**Additional file 1.** Clinical, historical and socio-environmental variables and presence of multidrug-resistant organisms stratified by age group.

**Additional file 2. **Sensitivity analysis of multiple logistic regression analysis excluding patients with *Aeromonas* spp. only isolated.

**Additional file 3.** Sensitivity analysis of multiple logistic regression analysis excluding patients with unknown prior medication use.

## Data Availability

The de-identified datasets used during the current study are available from the corresponding author on reasonable request.

## Abbreviations

AMR: Antimicrobial resistance
AST: Antimicrobial susceptibility testing
HIC: High-income country
LMIC: Low- and middle-income country
MDR: Multidrug resistance
MDRO: Multidrug-resistant organism
NIRUDAK: Novel, Innovative Research for Understanding Dehydration in Adults and Kids
TTGA: Taurocholate gelatin agar
WHO: World Health Organization
