# Supplementary material for: Multidrug-resistant enteric pathogens in older children and adults with diarrhea in Bangladesh: epidemiology and risk factors
Source: Trop Med Health. 2021 May 10;49:34. doi: 10.1186/s41182-021-00327-x (PMC8108363; doi:10.1186/s41182-021-00327-x)
Supplement: Supplementary file 1 — Additional file 1. Clinical, historical and socio-environmental variables and presence of multidrug-resistant organisms stratified by age group. [file 41182_2021_327_MOESM1_ESM.docx]

Additional File 1

File format: .docx

Title: Clinical, historical and socio-environmental variables and presence of multidrug-resistant organisms stratified by age group.

|  | Age Group | | | Overall  N=1,198  n (%) | p |
| --- | --- | --- | --- | --- | --- |
|  | Child  (5-17 years)  N=324  n (%) | Adult  (18-59 years)  N=498  n (%) | Elderly  (≥60 years)  N=376  n (%) |  |  |
| Female Sex | 119 (36.7) | 237 (47.6) | 179 (47.6) | 577 (48.2) | <0.01 |
| Altered Mental Status | 9 (2.8) | 32 (6.4) | 22 (5.9) | 63 (5.3) | 0.06 |
| Bloody Stool Reported | 7 (2.2) | 1 (0.2) | 5 (1.3) | 13 (1.1) | 0.03 |
| Mucoid Stool Reported | 78 (24.1) | 91 (18.3) | 74 (19.7) | 243 (20.3) | 0.12 |
| Abdominal pain | 143 (44.1) | 212 (42.6) | 160 (42.6) | 515 (43.0) | 0.89 |
| Vomiting (>3episodes/24hr) | 257 (79.3) | 360 (72.3) | 222 (59.0) | 839 (70.0) | <0.01 |
| Diarrhea Frequency |  |  |  |  | <0.01 |
| *≤ 10 episodes/24hr* | 136 (42.0) | 169 (33.9) | 100 (26.6) | 405 (33.8) |  |
| *>10 episodes/24hr* | 161 (49.7) | 252 (50.6) | 212 (56.4) | 625 (52.2) |  |
| *>20 episodes/24hr* | 27 (8.33) | 77 (15.5) | 64 (17.0) | 168 (14.0) |  |
| Prior Antibiotic Use | 101 (31.2) | 194 (39.0) | 140 (37.2) | 435 (36.3) | 0.07 |
| Highest Education Level |  |  |  |  | <0.01 |
| *No school* | 14 (4.3) | 111 (22.3) | 221 (59.8) | 346 (28.9) |  |
| *Primary School* | 156 (48.2) | 148 (29.7) | 103 (27.4) | 407 (34.0) |  |
| *Junior Secondary* | 90 (27.8) | 98 (19.7) | 22 (5.9) | 210 (17.5) |  |
| *Secondary +* | 64 (19.8) | 141 (28.3) | 30 (8.0) | 235 (19.6) |  |
| Water Source – Indoor Piped | 236 (72.8) | 358 (71.9) | 256 (68.1) | 850 (71.0) | 0.32 |
| Use of Treated Water | 147 (45.4) | 198 (39.8) | 153 (40.7) | 498 (41.6) | 0.26 |
| Non-Flush Toilet Use | 191 (59.0) | 291 (58.4) | 212 (56.4) | 504 (42.1) | 0.76 |
| Sick contacts at home | 82 (25.3) | 112 (22.5) | 74 (19.7) | 268 (22.4) | 0.20 |
| >9 ppl Sharing Waste Facilities | 176 (54.3) | 257 (51.6) | 152 (40.4) | 585 (48.8) | <0.01 |
| >5 ppl in Household | 121 (37.4) | 153 (30.7) | 123 (32.7) | 397 (33.1) | 0.14 |
| Time to hospital (>90 min) | 55 (17.0) | 109 (21.9) | 94 (25.0) | 258 (21.5) | 0.04 |
| Temperature (F), mean (SD) | 98.1 ± 1.28 | 97.62 ± 1.13 | 97.6 ± 1.17 | 97.7 ± 1.2 | <0.01 |
| Respiratory Rate (rpm), mean (SD) | 28.5 ± 5.2 | 28.7 ± 5.8 | 29.0 ± 6.1 | 28.7 ± 5.7 | 0.50 |
| Heart Rate (beats/min), mean (SD) | 117.5 ± 23.5 | 105.3 ± 19.9 | 94.6 ± 18.4 | 105.2 ± 22.3 | <0.01 |
| MAP (mmHg), mean (SD) | 69.6 ± 12.7 | 73.4 ± 16.4 | 74.2 ± 16.5 | 72.7 ± 15.6 | <0.01 |
| MUAC (mm), mean (SD) | 19.8 ± 3.5 | 25.2 ± 3.0 | 24.3 ± 2.5 | 234.4 ± 37.6 | <0.01 |
| % Dehydration, mean (SD) | 6.4 ± 3.2 | 5.6 ± 2.8 | 5.7 ± 3.2 | 5.9 ± 3.1 | <0.01 |
| Monthly Income ($100USD), mean (SD) | 1.7 ± 1.0 | 1.9 ± 1.4 | 2.0 ± 1.3 | 1.9 ± 1.3 | 0.01 |
| Presence of MDRO | 182 (56.2) | 265 (53.2) | 203 (54.0) | 650 (54.3) | 0.702 |
| *Abbreviations: MDRO, multidrug-resistant organisms; F, Fahrenheit; rpm, respirations/minute; USD, United States Dollars; MAP, mean arterial pressure; MUAC, mid-upper arm circumference*  *Figures are reported as n (%) unless otherwise specified* | | | | | |
