# Supplementary material for: Multidrug-resistant enteric pathogens in older children and adults with diarrhea in Bangladesh: epidemiology and risk factors
Source: Trop Med Health. 2021 May 10;49:34. doi: 10.1186/s41182-021-00327-x (PMC8108363; doi:10.1186/s41182-021-00327-x)
Supplement: Supplementary file 2 — Additional file 2. Sensitivity analysis of multiple logistic regression analysis excluding patients with Aeromonas spp. only isolated. [file 41182_2021_327_MOESM2_ESM.docx]

Additional File 2

Title: Sensitivity analysis of multiple logistic regression analysis excluding patients with *Aeromonas* spp. only isolated

| **Characteristic** | **aOR** | **95% CI** | **p** |
| --- | --- | --- | --- |
| Age (years) | 0.99 | 0.98-1.00 | 0.11 |
| Female Sex | 1.20 | 0.88-1.63 | 0.25 |
| Temperature (F) | 0.74 | 0.63-0.87 | <0.01 |
| Respiratory Rate (breaths/min) | 0.98 | 0.96-1.01 | 0.26 |
| Heart Rate (beats/min) | 0.99 | 0.99-1.00 | 0.12 |
| Mean Arterial Pressure (mmHg) | 0.99 | 0.98-1.00 | 0.19 |
| Mid-upper arm circumference (cm) | 0.99 | 0.99-1.00 | 0.02 |
| % Dehydration | 0.99 | 0.94-1.05 | 0.78 |
| Altered Mental Status | 0.56 | 0.28-1.12 | 0.10 |
| Bloody Stool Reported | 3.86 | 0.88-17.02 | 0.07 |
| Mucoid Stool Reported | 1.14 | 0.78-1.67 | 0.49 |
| Abdominal pain | 0.97 | 0.71-1.31 | 0.83 |
| Vomiting (>3episodes/24hr) | 0.98 | 0.70-1.38 | 0.90 |
| Diarrhea Frequency |  |  | 0.34 |
| *≤ 10 episodes/24hr* | - | - |  |
| *>10 episodes/24hr* | 1.13 | 0.81-1.58 |  |
| *>20 episodes/24hr* | 1.43 | 0.89-2.32 |  |
| Prior Antibiotic Use | 1.86 | 1.36-2.55 | <0.01 |
| Monthly Household Income ($100USD) | 1.00 | 0.88-1.13 | 0.96 |
| Highest Education Level |  |  | 0.75 |
| *No school* | - | - |  |
| *Primary School* | 1.15 | 0.74-1.79 |  |
| *Junior Secondary* | 1.23 | 0.72-2.09 |  |
| *Secondary +* | 0.98 | 0.58-1.66 |  |
| Water Source – Indoor Piped | 0.93 | 0.65-1.33 | 0.68 |
| Use of Treated Water | 1.04 | 0.75-1.45 | 0.82 |
| Non-Flush Toilet Use | 1.33 | 0.98-1.80 | 0.07 |
| Sick contacts at home | 1.05 | 0.74-1.49 | 0.78 |
| >5 ppl in Household | 1.34 | 0.98-1.84 | 0.07 |
| Time to hospital (>90 min) | 1.46 | 1.01-2.12 | 0.049 |
| *Abbreviations: OR, odds ratio; CI, confidence interval; USD, United States Dollar*  *- : reference level* | | | |
