# Supplementary material for: Multidrug-resistant enteric pathogens in older children and adults with diarrhea in Bangladesh: epidemiology and risk factors
Source: Trop Med Health. 2021 May 10;49:34. doi: 10.1186/s41182-021-00327-x (PMC8108363; doi:10.1186/s41182-021-00327-x)
Supplement: Supplementary file 3 — Additional file 3. Sensitivity analysis of multiple logistic regression analysis excluding patients with unknown prior medication use. [file 41182_2021_327_MOESM3_ESM.docx]

Additional File 3

File format: .docx

Title: Sensitivity analysis of multiple logistic regression analysis excluding patients with unknown prior medication use

| **Characteristic** | **aOR** | **95% CI** | **p** |
| --- | --- | --- | --- |
| Age (years) | 1.00 | 0.99-1.00 | 0.24 |
| Female Sex | 1.21 | 0.92-1.59 | 0.18 |
| Temperature (F) | 0.89 | 0.78-1.02 | 0.09 |
| Respiratory Rate (breaths/min) | 0.98 | 0.95-1.00 | 0.06 |
| Heart Rate (beats/min) | 1.00 | 0.99-1.00 | 0.16 |
| Mean Arterial Pressure (mmHg) | 0.99 | 0.99-1.00 | 0.30 |
| Mid-upper arm circumference (cm) | 1.00 | 0.99-1.00 | 0.25 |
| % Dehydration | 0.96 | 0.92-1.01 | 0.11 |
| Altered Mental Status | 0.75 | 0.41-1.36 | 0.34 |
| Bloody Stool Reported | 3.94 | 0.80-19.40 | 0.09 |
| Mucoid Stool Reported | 0.94 | 0.67-1.32 | 0.70 |
| Abdominal pain | 0.94 | 0.72-1.23 | 0.65 |
| Vomiting (>3episodes/24hr) | 0.95 | 0.70-1.28 | 0.73 |
| Diarrhea Frequency |  |  | <0.01 |
| *≤ 10 episodes/24hr* | - | - |  |
| *>10 episodes/24hr* | 1.47 | 1.10-1.97 |  |
| *>20 episodes/24hr* | 2.08 | 1.34-3.23 |  |
| Prior Antibiotic Use | 1.74 | 1.32-2.29 | <0.01 |
| Monthly Household Income ($100USD) | 1.02 | 0.92-1.14 | 0.70 |
| Highest Education Level |  |  | 0.55 |
| *No school* | - | - |  |
| *Primary School* | 1.10 | 0.76-1.61 |  |
| *Junior Secondary* | 1.13 | 0.71-1.80 |  |
| *Secondary +* | 0.86 | 0.54-1.36 |  |
| Water Source – Indoor Piped | 1.03 | 0.74-1.42 | 0.88 |
| Use of Treated Water | 1.06 | 0.79-1.42 | 0.71 |
| Non-Flush Toilet Use | 1.52 | 1.15-2.00 | <0.01 |
| Sick contacts at home | 1.31 | 0.95-1.81 | 0.10 |
| >5 ppl in Household | 1.09 | 0.82-1.46 | 0.54 |
| Time to hospital (>90 min) | 1.41 | 1.01-1.97 | 0.04 |
| *Abbreviations: OR, odds ratio; CI, confidence interval; USD, United States Dollar*  *- : reference level* | | | |
